# Comprehensive Analysis of the Prognostic Marker and Immune Infiltrates of LDLR-Related Proteins Family Members in Breast Cancer

**DOI:** 10.30699/IJP.2024.1995769.3077

**Published:** 2024-03-29

**Authors:** Shabnam Shahidi, Parvin Ansari Shayesteh, Mahsa Alami, Negin Parsamanesh

**Affiliations:** 1 *Department of Genetics and Molecular Medicine, School of Medicine, Zanjan University of Medical Sciences, Zanjan, Iran *; 2 *Metabolic Diseases Research Center, Zanjan University of Medical Sciences, Zanjan, Iran*; a * these authors contributed equally*

**Keywords:** Breast Cancer, LDLR-related protein (LRP), Wnt signaling pathway

## Abstract

**Background & Objective:**

Breast cancer (BC) is one of the most frequent tumors worldwide, accounting for 15% of all cancer-related deaths. A timely diagnosis of BC is essential for optimal treatment and increasing patients' survival rates. LRP family proteins are important components of cell-surface receptors involved in numerous biological activities. Expression of LRP is related to breast malignancy. In this study, we initially studied the expression of LRPs in BC tissues compared to normal tissues—the relation of LRP expression with relapse-free survival (RFS) and overall survival (OS). Then, we investigated the association of LRPs relation and immune infiltrating abundance.

**Methods:**

We analyzed the LDLR family expression and prognostic value in BC by mining UALCAN, TIMER, and Kaplan-Meier plotter databases. Subsequently, we explored the association of LDLR expression and immune infiltrating abundance via the TIMER database.

**Results:**

Expression levels of LRP1/2/4/9/10 were found to be higher in the cases with positive estrogen receptors. There was a positive association between LRP1/6 expression and the infiltration of CD8+ T cells, CD4+ T Cell, Macrophage, Dendritic Cell, and Neutrophil.

**Conclusion:**

Our study recommends LDLR as a potential prognostic biomarker that can be promising to improve the survival of BC patients' survival. However, further investigations are needed to evaluate the studied LDLR members in more detail.

## Introduction

Breast cancer (BC) is a heterogeneous disease that is caused by the accumulation of genetic aberrations (1). Most BCs occur sporadically, and germline mutations are seen in 10% of all cases (2). Based on the American Cancer Society (ACS), BC is one of the two leading causes of women's death, with a mortality rate of 15%, as well as the first and most common malignancy among women. The incidence of female breast cancer is rising by approximately 0.5% yearly, and it is mainly diagnosed in the fourth decade of life (3). In addition, Iranian studies reveal the same statistics except in the multi-dimensional aspect of BC(4).

The gene family known as low-density lipoprotein receptor (LDLR) encodes fourteen receptors characterized by their transmembrane structure. These receptors are commonly referred to as LDLR-related proteins (LRPs) and encompass LDLR, VLDLR, LRP1 (also known as CD91/A2MR), LRP1B, LRP2 (also known as megalin/GP330), LRP3, LRP4 (also known as MEGF7), LRP5, LRP6, LRP8 (also known as ApoER2), LRP10 (also known as LRP9), LRP11 (also known as SorLA), LRP12 (also known as ST7), and LRAD3 (5). This family consists of an extracellular chain and a short cytoplasmic domain, with a motif (e.g., cysteine-rich complement-type repeats). This motif is involved in triggering the ligand-dependent endocytosis mechanism. They have inhibitory and signaling functions involved in multiple physiopathological processes such as neurobiology, vascular integrity, and cancer progress (6-8). LRPs also have multiple functions, such as growth factor signaling, matricellular proteins, cellular transformation, cell-matrix adhesion revenue, and chemoattraction inflammation. As a result, they affect the tumor cells and their microenvironment (5, 9). Today, LRPs are considered a molecular target of breast cancer. LRP receptor expression is high in breast cancer and causes more LDL absorption from the blood. It should be noted that cancer cells consume more cholesterol than normal cells. Many studies describe the relationship between LRP receptors and various cancers such as breast, prostate, and colorectal. In addition, the mutation in the LRP receptor gene affects its multiple functions such as binding, synthesis, transport, and internalization (10). Each member of this family has different roles that may play a role in the occurrence of different cancers such as breast cancer, of course, their roles have not been fully identified (11). 

Therefore, this study aims to provide more insight into the function of some members of the LRP family and their potential roles in breast cancer. Therefore, we first studied the LRPs prognostic and expression value in BC by Kaplan-Meier plotter, UALCAN, bc-GenExMiner, and TIMER databases. Then, we investigated the relation between LRP expression and immune infiltrating abundance with TIMER databases.

## Material and Methods

**Kaplan-Meier Plotter**

The prognostic value of the mRNA expression of LRP family members in breast cancer patients was evaluated by the Kaplan-Meier plotter (http://kmplot.com/analysis) (18). Subsequently, survival analysis was done for relapse-free survival (RFS) and overall survival (OS) in all BC patients. The cutoff for meaning was determined as a log-rank P-value of less than 0.05 (12).

**bc-GenExMiner v4.8**

The bc-GenExMiner v4.8 (http://bcgenex.ico.unicancer.fr/BC-GEM/) (13 was employed to ascertain the correlation between the expression of LRPs and BC clinicopathologic factors, such as tissue nodal status, age, progesterone receptor (PR), estrogen receptor (ER), and human epidermal growth factor 2 (HER2). The disparity in mRNA expression of LRPs among BC patients with diverse molecular and clinical factors was assessed using Dunnett-Tukey-Kramer's and Welch's tests, with a meaning level of P-value < 0.05 (13).

**GEPIA**

GEPIA, a web-based analysis tool accessible at http://gepia.cancer-pku.cn/, is a repository of RNA sequence expression data for both tumor and normal tissue samples. This tool was created by the esteemed institution of Peking University. We have conducted an examination of the differential expression of mRNA in the LRP family across tumor and normal tissues by utilizing GEPIA. A p-value cutoff of 0.05 was employed to ensure remarkable remarkable (14). 

**UALCAN**

The UALCAN tool was used to examine the relative transcriptional expression of the LRP family in various stages of breast cancer. This database utilizes data from The Cancer Genome Atlas (TCGA) for its analyses. Any p-value less than 0.05 was deemed statistically remarkable (15).

**STRING**

STRING (https://string-db.org/), an online resource, possesses the capability to accumulate, evaluate, and merge diverse sources of protein-protein interaction (PPI) data that are openly accessible. Additionally, this platform offers computational forecasts regarding potential functions to enhance the data above. Consequently, we showed a comprehensive PPI network analysis of LRP family constituents and their corresponding ligands (16).

**TIMER**

The TIMER tool (https://cistrome.shinyapps.io/timer/) was used in order to validate the relation between the LRPs expression and the presence of immune infiltrating cells, namely B cells, CD8+ T cells, CD4+ T cells, neutrophils, macrophages, and Dendritic Cell (17). 

**GeneMANIA**

GeneMANIA, a comprehensive online repository accessible at http://www.genemania.org, serves as a valuable dataset that furnishes data pertaining to protein and genetic interactions, intricate pathways, co-expression patterns, co-localization relationships, and protein domain similarity concerning the genes that are submitted. We successfully employed this database to construct a protein–protein interaction network encompassing eleven LRP class members and their respective ligands (18). 

**Metascape**

Metascape (http://metascape.org) is a reliable platform utilized to annotate genes and conduct gene list enrichment analysis. In this particular investigation, the "Express Analysis" module was employed to validate the LRP class enrichment and their closely associated neighboring genes according to the functional annotation of gene lists. The "Functional enrichment analysis" was also performed on the LRP members, encompassing the KEGG pathway and GO (comprising molecular functions, cellular components, and biological processes). Notably, a P-value of less than 0.05 was established as the threshold for remarkable (19). 

**Human Protein Atlas**

The online platform that encompasses immunohistochemistry-derived expression data known as Human Protein Atlas (https://www.proteinatlas.org/about/licence) integrates diverse omics technologies, including mass spectrometry-based proteomics, antibody-based imaging, transcriptomics, and system biology. In our investigation, we employed immunohistochemical imaging to directly compare protein expression levels of distinct members of the LRPs family in normal and BC tissues (20).

## Results

**The LRP Family Prognostic Value in BC Patients**

**Fig. 1:**
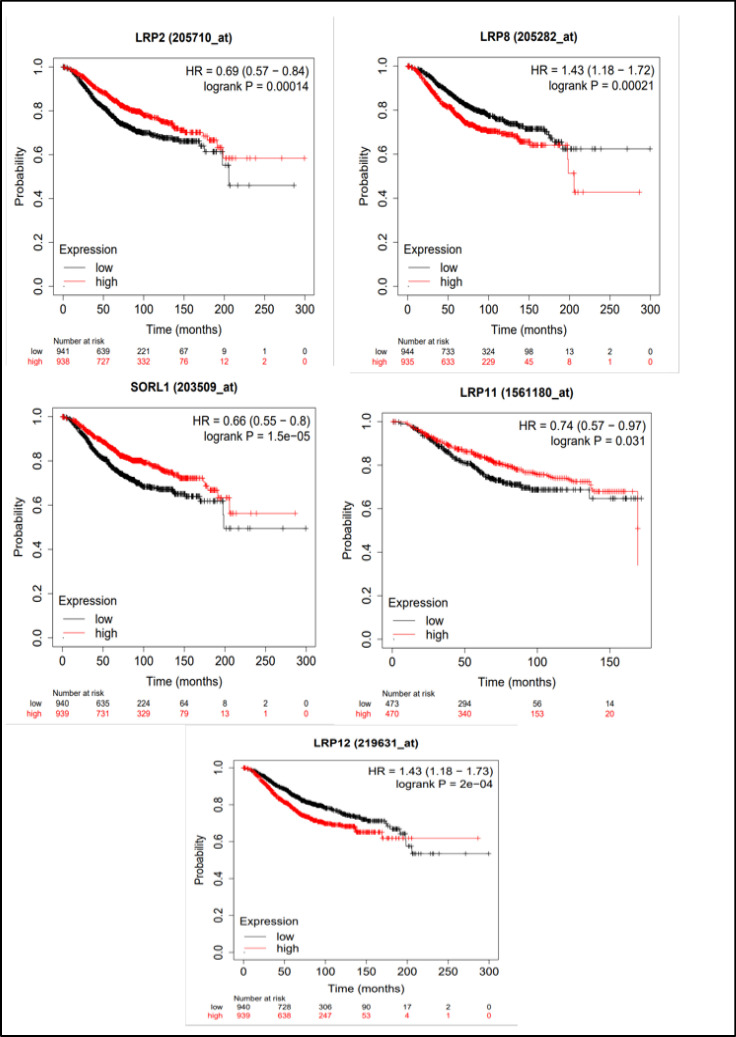
The relationship of mRNA expression of LRP2/8/9/11/12 with overall survival (OS) of the BC cases (Kaplan-Meier Plotter)

**Fig. 2:**
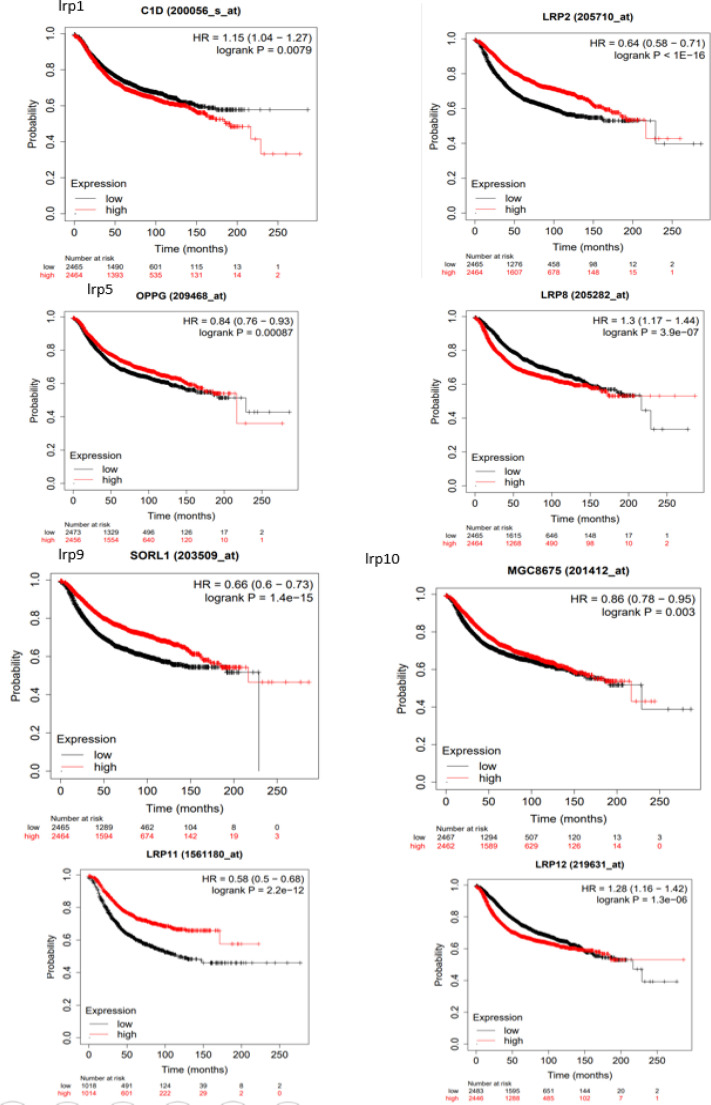
The association of LRP1/2/5/8/9/10/11/12 mRNA expression with relapse-free survival (RFS) of the BC cases (Kaplan-Meier Plotter)

Initially, we studied the prognostic significance of LRPS family members in all patients with BC. The elevated mRNA expression of LRP 2/9/11 exhibited a clear correlation with favorable overall survival (OS) in all BC patients. On the other hand, heightened expressions of LRP 8/12 were distinctly linked to unfavorable OS (Figure 1). Subsequently, we evaluated the prognostic value of LRPS family members in all BC patients. The increased mRNA expression of LRP 2/5/9/10/11 was significantly related to favorable relapse-free survival (RFS) in all BC patients. However, elevated expressions of LRP 1/8/12 were unequivocally associated with unfavorable RFS (Figure 2).

**Relationship of LRP mRNA Stages with Clinicopathological Characters in BC Patients**

**Fig. 3:**
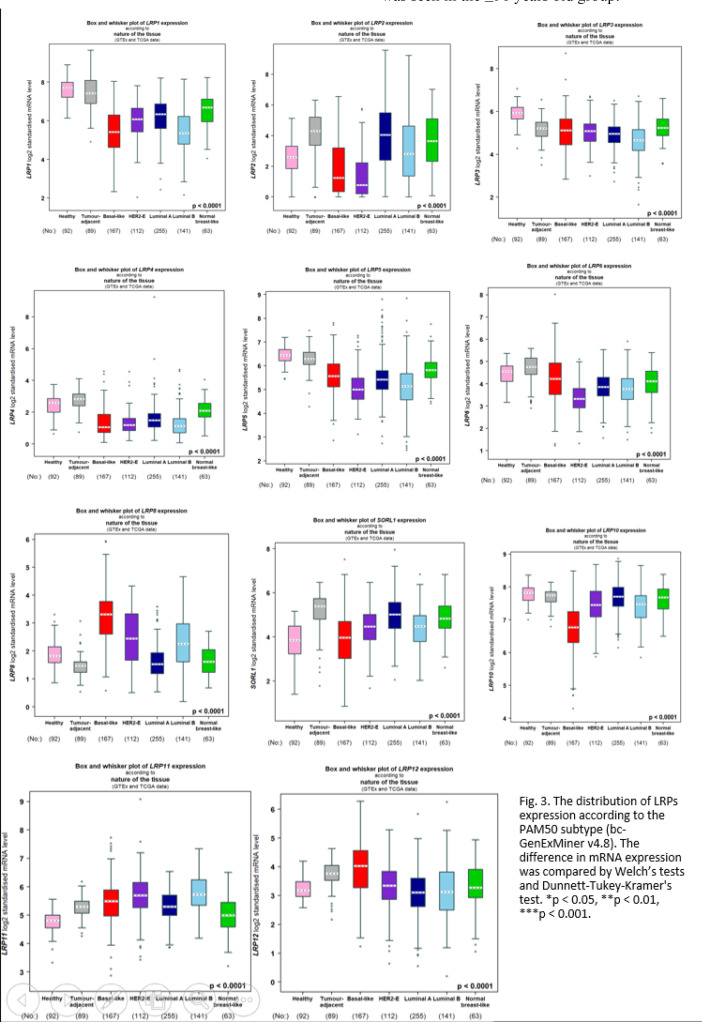
The distribution of LRPs expression according to the PAM50 subtype (bc-GenExMiner v408). The difference in mRNA expression was compared using Welch's tests and Dunnet-Tukey-Kramer's test. *P<0.05, **P<0.01, ***P<0.001

We further recognized the relation of mRNA expression of distinct LRPs with clinicopathological BC characters. As shown in Figure 3, we found an upregulated expression of LRP2/9/10 in the >51 years old group compared to that in the ≤51 years old group (*P*<0.05), while upregulated expression of LRP4/8/12 was seen in the ≤51 years old group.

The mRNA level of LRP1/4/10 was higher in the positive lymph nodes, while in the negative lymph nodes of patients with BC, the mRNA level of LRP 6/8 was found to be higher (*P*<0.05). According to Table 1, compared to the luminal subtype, the mRNA levels of LRP2/4/5/6 were elevated in HER2-negative (*P*<0.001). Among them, expression of LRP3/5/6/8/12 was higher in the basal-like subtype and TNBC subtype (*P*<0.001). However, the expression of LRP1/2/4/9/10 was higher in the non-basal-like subtype and non-TNBC subtype (*P*<0.001).

Expression levels of LRP1/2/4/9/10 were higher in those cases with positive estrogen receptors (ER+) (*P*<0.001), while the mRNA level of LRP3/6/8/12 was higher in the cases with negative estrogen receptors (ER-). Expression levels of LRP1/2/9/10 were higher in the cases with positive progesterone receptors (PR+), while cases with negative progesterone receptors (PR-) presented with a higher mRNA level of LRP3/6/8/12 (*P*<0.001).

**Fig. 4:**
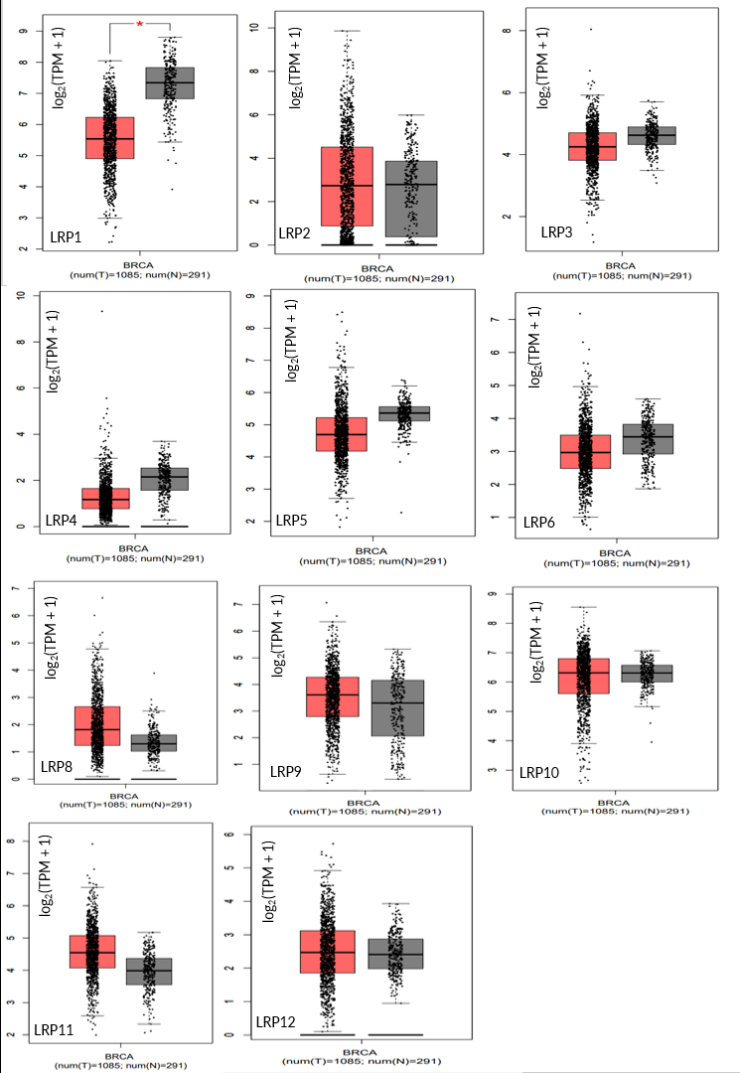
The box plot expression of LROs in BC. The box color of red indicates normal. The method for differential analysis is one-way-ANOVA, using disease state as a variable for calculating differential expression and asterisk means statistically significant, with each dot representing a distinct tumor or normal sample (GEIPA Database; TPM: Transcripts Per Million; T: Tumor; N: Normal)

As shown in Figure 4, mRNA expression of LRP 1 in normal tissue was found to be higher than in the tumoral tissue (*P*<0.01).

**Fig. 5:**
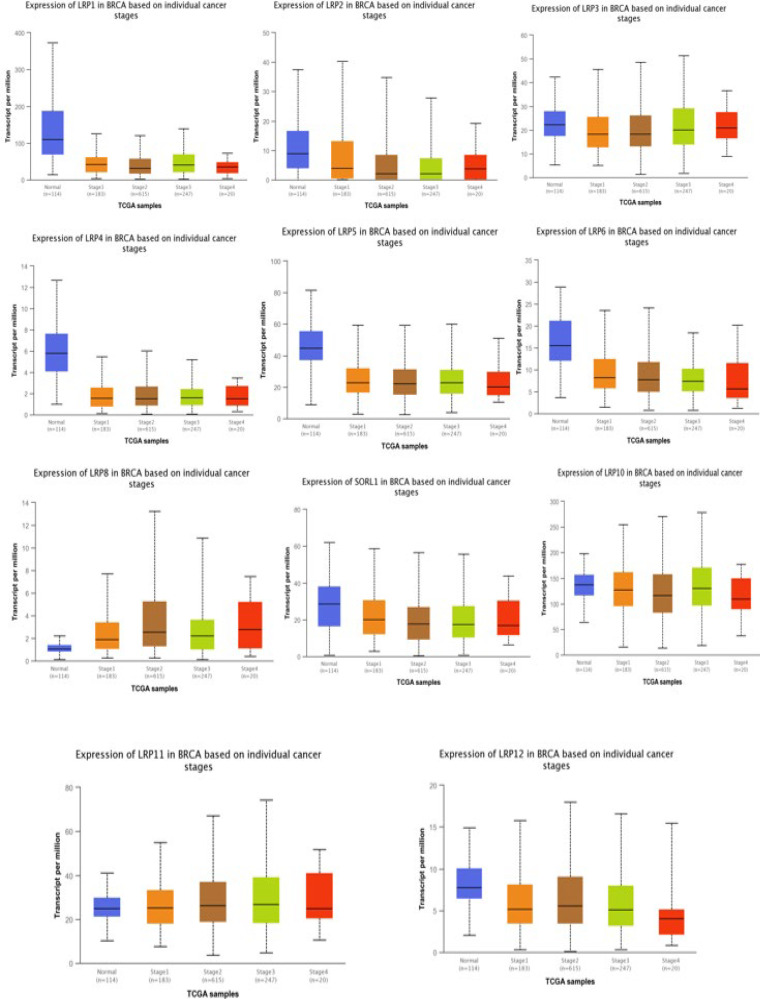
The mRNA expression of distinct LRP family members based on the individual tumor stages and normal breast tissues (ualcan)

Expression of LRP1 in normal tissue showed significantly a higher level than the four stages of the cancer (*P*<0.00001). LRP4 Expression in normal tissue was remarkably higher than in stages 2, 3, and 4 of the tumor (*P*<0.02). The expression level of LRP5/LRP9 in normal tissue revealed a notably higher level compared to those in stages 1/2/3 of the cancer (*P*<0.001). LRP6 expression in normal tissue was meaningfully higher than in stages 1, 2, and 3 of cancer, and in stages 1 and 2, it was outstandingly higher than stage 3 (*P*<0.0005). |The mRNA level of LRP8 in the four stages of the cancer was considerably higher than in normal tissue, and in stage 2, it was particularly higher than in stage 1 (*P*<0.0005). LRP10 expression in normal tissue and stage 3 was significantly higher than stage 2 (*P*<0.0005). The expression level of LRP11 in stages 1, 2, and 3 of cancer was notably higher than in normal tissue, and in stages 2 and 3 of cancer, it was remarkably higher than stage 1 (*P*<0.001). The expression of LRP12 in normal tissue was meaningfully higher than in stages 1 and 3 of cancer, and in stage 2, it was seriously higher than in stage 3 (*P*<0.001) (Figure 5). 

**Immune Cell Infiltration LRPs in Patients With BC**

**Fig. 6:**
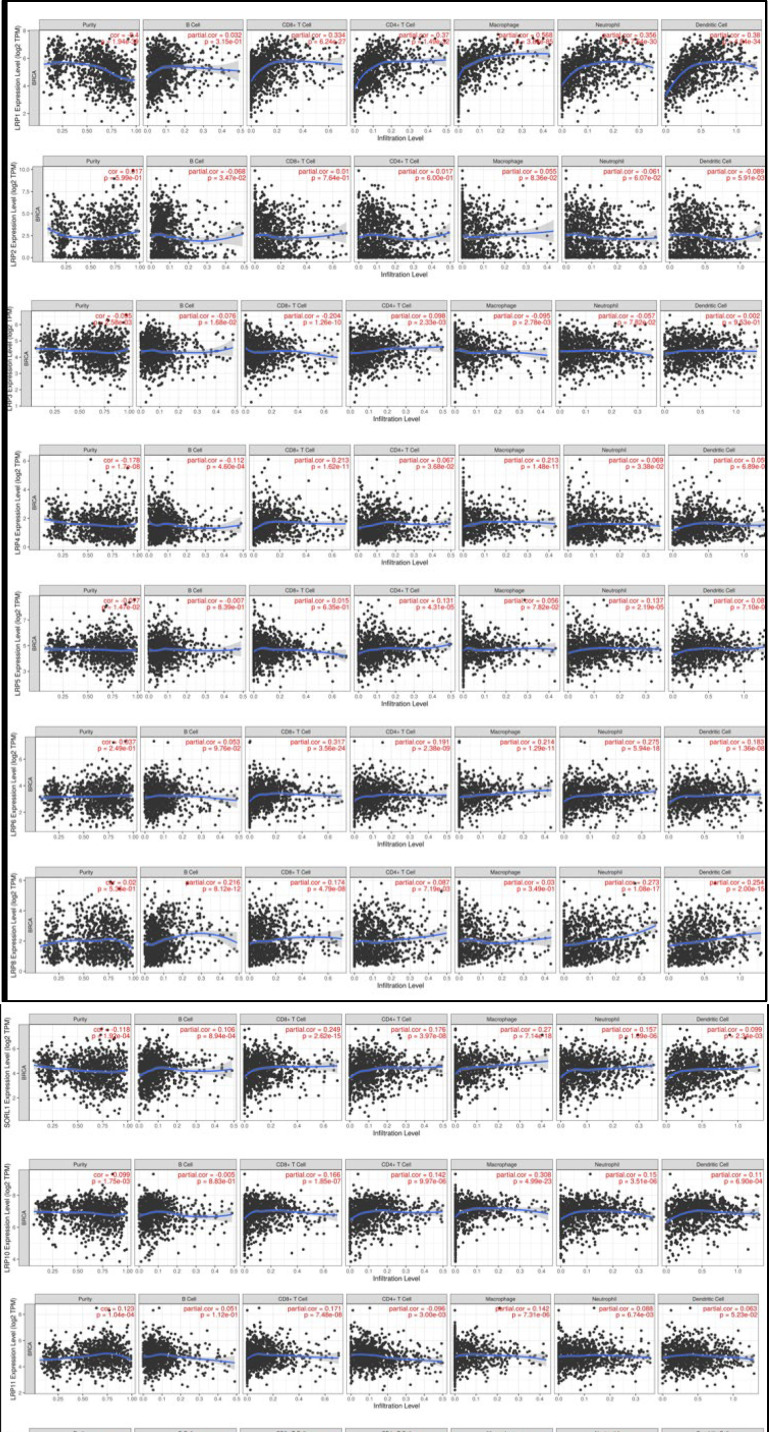
Correlation of distinct LRPS expression with B cell, CD4+ T Cell, CD8+ T Cell, neutrophil, macrophage, and dendritic cell expressions in the BC (TIMER)

We explored the association between differentially expressed LRPs and immune cell infiltration by the TIMER database. There was a positive association between LRP1/6 expression and infiltration of the CD8+ T cells, CD4+ T Cell, macrophage, dendritic cell, and neutrophil. LRP2 expression was negatively associated with the B cells and dendritic cell infiltration. LRP3 expression was negatively associated with infiltration of the macrophages, CD8+ T cells, and B cells and positively with infiltration of the CD4+ T cells. LRP4 expression was negatively related to infiltration of the B cells and positively with infiltration of the CD8+ T cells, CD4+ T cells, macrophages, and Neutrophils. There was a positive relation between expression of the LRP5 and infiltration of the CD4+ T Cell and neutrophil. There was a positive relation between the expression of LRP 6/8/9/10/12 and infiltration of the dendritic Cells. There was a positive relation between expression of LRP8/9/10/12 and infiltration of the CD8+ T cells, CD4+ T Cells, and neutrophils. There was a positive relation between expressions of LRP8/9 and infiltration of the B cells. There was a positive relation between the expression of LRP9/10/12 and infiltration of the macrophages. LRP11 expression exhibited a negative correlation with CD4+ T cells while displaying a positive correlation with CD8+ T cells, macrophages, and neutrophils (Table 2, Figure 6).

**Table 1:** The correlation between mRNA levels of LRPs and clinical pathological features of the BC patients (BC gene examiner)

Criteria	LRP1			LRP2			LRP3			LRP4			LRP5			LRP6		
age	**no**	**mRNA**	**P-value**	**no**	**mRNA**	**P-value**	**no**	**mRNA**	**P-value**	**no**	**mRNA**	**P-value**	**no**	**mRNA**	**P-value**	**no**	**mRNA**	**P-value**
>51	476		0.1283	476	↑	0.0006	476		0.0012	476		0.0012	476		0.5282	476		0.9623
<51	267			267			267	↑		267			267			267		
Nodal status																		
(-)	332		0.0215	332		0.9934	332		0.003	332		0.003	332	↑	0.3611	332		0.0358
(+)	358	↑		358			358	↑		358			358			358		
ER/PR(HC)																		
ER+/PR+	456		0.0001	456	↑	0.0001	456	↓	0.038	456		0.038	456		0.0026	456		0.053
ER+/PR-	71			71			71	↑		71			71			71		
ER-/PR+	14	↑		14			14			14			14	↓		14	↑	
ER-/PR-	171	↓		171	↓		171			171	↑		171	↑		171	↑	
HER2+	109		0.8348	109		0.0001	109		0.0243	109		0.0243	109		0.0032	109		0.0001
HER2-	396			396	↑		396	↑		396	↑		396	↑		396		
Basal like and TNBC																		
Not	552	↑	0.0001	552	↑	0.0001	552	↑	0.003	552		0.003	552		0.005	552		0.0001
Basal like/TNBC	71			71			71			71	↑		71	↑		71	↑	

**Table 2:** LRP family members that correlate with B cell, CD8+ T Cell, CD4+ T Cell, macrophage, neutrophil, and dendritic cell expressions in BC (TIMER)

Cancer	Variable	Partial. Correlation	P-value
BRCA-lrp01	CD4+ T Cell	0.370	1.486e-32
	CD8+ T Cell	0.334	6.243e-27
	Dendritic Cell	0.380	4.844e-34
	Macrophage	0.568	3.876e-85
	Neutrophil	0.356	7.544e-30
BRCA-lrp02	B Cell	-0/068	0.035
	Dendritic Cell	-0/09	0.006
BRCA-lrp03	B Cell	-0/08	0.017
	CD4+ T Cell	0.098	0.002
	CD8+ T Cell	-0/204	1.264e-10
	Macrophage	-0/095	0.003
BRCA-lrp04	B Cell	-0/111	0.001
	CD4+ T Cell	0.067	0.037
	CD8+ T Cell	0.213	1.618e-11
	Macrophage	0.213	1.480e-11
	Neutrophil	0.069	0.034
BRCA-lrp05	CD4+ T Cell	0.131	4.307e-05
	Dendritic Cell	0.087	0.007
	Neutrophil	0.137	2.191e-05
BRCA-lrp06	CD4+ T Cell	0.191	2.378e-09
	CD8+ T Cell	0.317	3.563e-24
	Dendritic Cell	0.183	1.362e-08
	Macrophage	0.214	1.286e-11
	Neutrophil	0.275	5.940e-18
BRCA-lrp08	B Cell	0.216	8.122e-12
	CD4+ T Cell	0.087	0.007
	CD8+ T Cell	0.174	4.791e-08
	Dendritic Cell	0.254	2.002e-15
	Neutrophil	0.273	1.080e-17
BRCA-lrp09	B Cell	0.106	0.001
	CD4+ T Cell	0.176	3.968e-08
	CD8+ T Cell	0.250	2.624e-15
	Dendritic Cell	0.098	0.002
	Macrophage	0.270	7.140e-18
	Neutrophil	0.157	1.092e-06
BRCA-lrp10	CD4+ T Cell	0.142	9.969e-06
	CD8+ T Cell	0.166	1.850e-07
	Dendritic Cell	0.110	0.001
	Macrophage	0.308	4.986e-23
	Neutrophil	0.150	3.511e-06
BRCA-lrp11	CD4+ T Cell	0/095	0.003
	CD8+ T Cell	0.171	7.479e-08
	Macrophage	0.142	7.309e-06
	Neutrophil	0.088	0.007
BRCA-lrp12	CD4+ T Cell	0.126	9.362e-05
	CD8+ T Cell	0.319	1.340e-24
	Dendritic Cell	0.252	2.666e-15
	Macrophage	0.278	6.595e-19
	Neutrophil	0.283	5.247e-19

**Neighbor Gene Network and Interaction Studies of LRPs in Patients with BC**

**Fig. 7:**
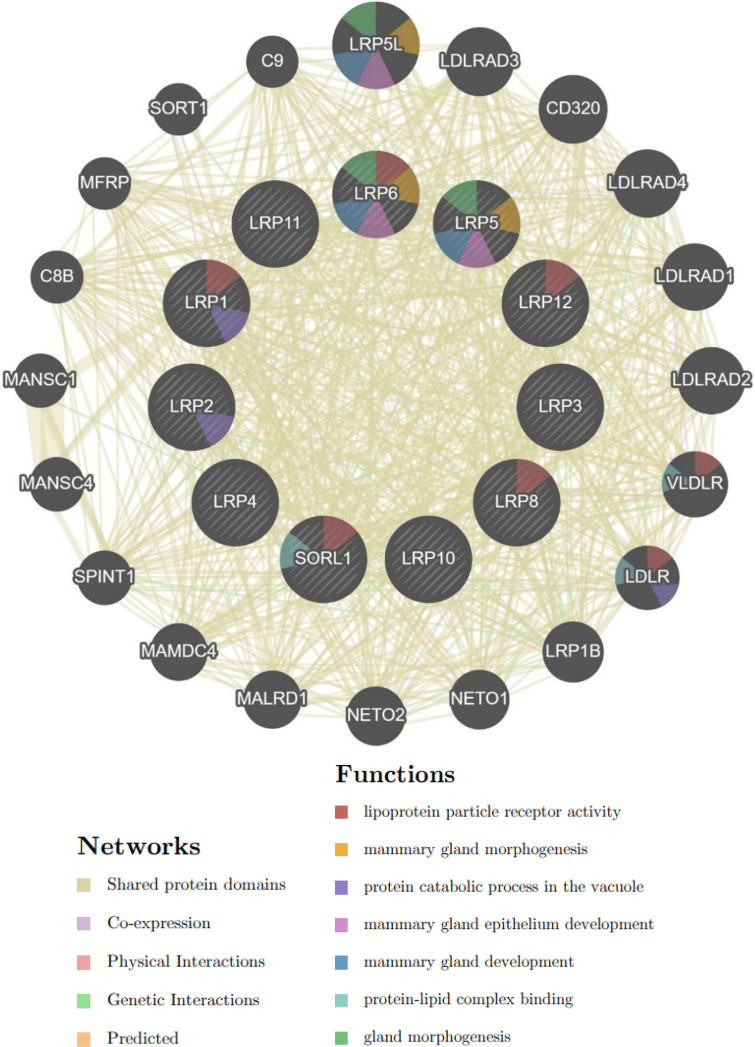
Protein–protein interaction network of eleven LRP family members and their ligands by GeneMANIA databases

**Fig. 8:**
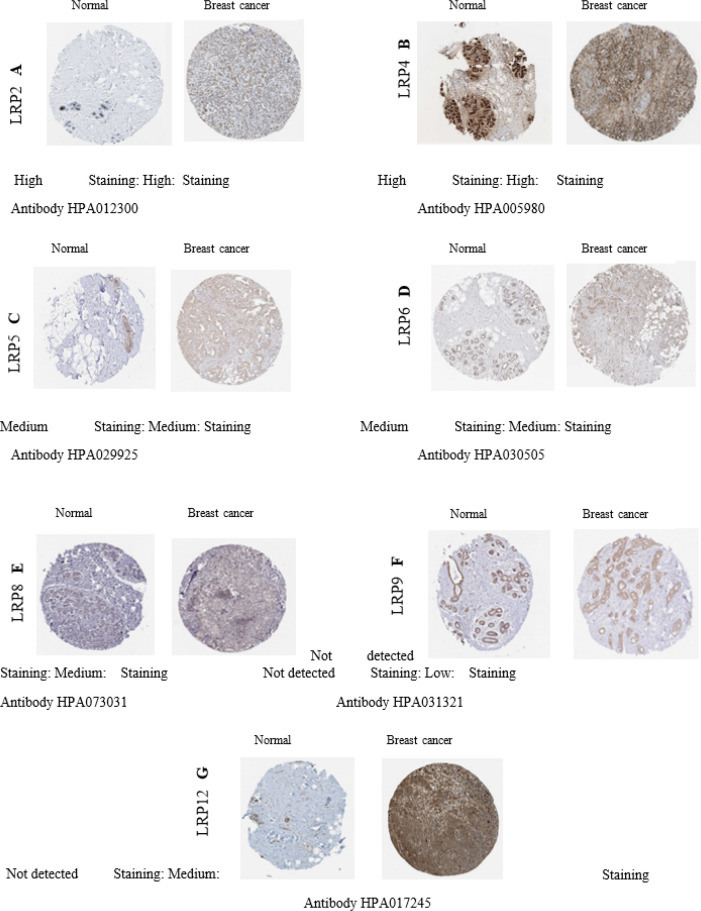
Representative Immunohistochemical images of distinct members from the LRPs family in the breast cancer, as per the Human Protein Atlas Database. Scale bars, 200 μm

Members of the LRP family were related to the membrane proteins and receptors (Figures 7 and 8).

**Fig. 9:**
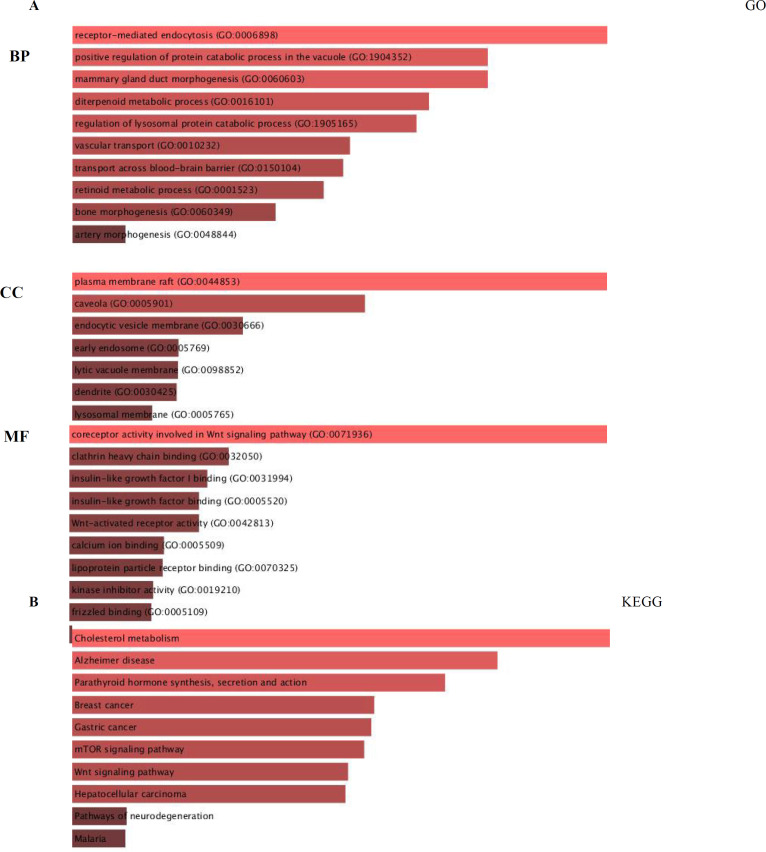
The enrichment study of differentially expressed LRP families and frequently altered neighboring genes in Metascape. **(A)** Bar plot of GO enrichment in cellular component terms (CC), biological process terms (BP), and molecular function terms (MF). **(B)** Bar plot of KEGG enriched terms

We analyzed LRP protein expression patterns in BC using the HPA database. The outcome shows that high protein expressions of LRP2/4 were detected in normal breast tissue and cancerous ones. Furthermore, medium protein expressions of LRP5/6 were expressed in both normal and cancer tissues. However, LRP9/12 medium expressions and LRP8 low expression were detected in BC tissues, whereas LRP8/9/12 was not expressed in normal breast tissues (Figure 8). No expression of LRP1/3/10/11 was observed in normal and tumor tissues. Our results showed that transcription and protein expression of LRPs were overexpressed in BC compared to normal breast tissues. The enrichment study of differentially expressed LRP families frequently could alter neighboring genes in Metascape (Figure 9).

**Prediction of miRNA and TF-Associated with the LRP Members**

Transcription factors (TFs) and miRNAs possibly controlling LRP members, were retrieved from miRTarBase and ChEA databases and were reviewed in Tables 3 and 4, respectively. LRP1, LRP2, LRP3, LRP4, LRP5, LRP6, LRP8, LRP9, LRP10, and LRP12 were included in Enrichr. We found that thirteen transcription factors (P300, NFE2L2, KDM2B, SA1, GATA6 FOXA1, DPY30, TCF7, UBF1/2, HNF4A, CDX2, SPI1, and TCF4) were related with the regulation of LRP families (Table 3). However, among the 23 highly enriched in the miRTarBase, hsa-miR-5011-5p, hsa-miR-5571-5p, hsa-miR-29b-3p, hsa-miR-516a-5p, hsa-miR-628-3p were the five most highly upregulated in LRP families (Table 4).

**Predictable Functions and Pathways of LRPs in Breast Cancer**

Among the 203 extremely enriched functions in the BP category, receptor-mediated endocytosis, positive regulation of protein catabolic progression in the vacuole, mammary gland duct morphogenesis, diterpenoid metabolic process, regulation of lysosomal protein catabolic process were found to be the five most factors related to the tumorigenesis and breast cancer progression. The five most extremely enriched items in the CC category included plasma membrane raft, caveola, endocytic vesicle membrane, early endosome, and lytic vacuole membrane. In the molecular function of the MF class, differentially expressed LRP families and their neighboring genes were generally enriched in coreceptor activity involved in the Wnt signaling pathway, clathrin heavy chain binding, insulin-like growth factor binding, insulin-like growth factor I binding, and Wnt-activated receptor actions. KEGG pathway studies were also done. Obviously, among the top ten KEGG pathways, cholesterol metabolism, Alzheimer's disease, parathyroid hormone synthesis, BC, and gastric cancer were shown to be significantly associated with LRP families. Protein-protein interaction network of eleven LRP family members is shown in Table 5. 

**Table 3:** The key regulated factor of LRP families in the BC (ChEA 2016)

TF	Description	Regulated Genes	P-value
P300	E1A Binding Protein P300	LRP5; LRP4; LRP10; LRP8; LRP11	0.003
NFE2L2	NFE2 Like BZIP Transcription Factor 2	LRP2; LRP8; LRP12	0.017
KDM2B	Lysine Demethylase 2B	LRP5; LRP4; LRP3; LRP8	0.018
SA1	Stromal Antigen 1	LRP1; LRP4; LRP2; LRP8	0.018
GATA6	GATA Binding Protein 6	LRP5; LRP2; LRP10; LRP8	0.018
FOXA1	Forkhead Box A1	LRP1; LRP3; LRP10; LRP12	0.018
DPY	Dpy-30 Histone Methyltransferase Complex Regulatory Subunit	LRP1; LRP4; LRP2; LRP11	0.018
TCF7	Transcription Factor 7	LRP10; LRP8;LRP12;LRP6	0.018
UBF1/2	Upstream Binding Transcription Factor	LRP5;LRP4;LRP10;LRP8	0.018
HNF4A	Hepatocyte Nuclear Factor 4 Alpha	LRP1;LRP5;LRP2	0.021
CDX2	Caudal Type Homeobox 2	LRP5;LRP6	0.030
SPI1	Spi-1 Proto-Oncogene	LRP10;LRP11;LRP6	0.036
TCF4	Transcription Factor 4	LRP4;LRP2;LRP8;LRP12;LRP6	0.042

**Table 4:** The key regulated MiR of LRP families in the BC (miRTarBase 2017 ).

	Term	Overlap	P-value	Regulated gene
1	hsa-miR-5011-5p	3/653	0.005	LRP8;LRP12;LRP6
2	hsa-miR-5571-5p	2/217	0.006	LRP10;LRP6
3	hsa-miR-29b-3p	2/261	0.009	LRP10;LRP6
4	hsa-miR-516a-5p	1/18	0.010	LRP10
5	hsa-miR-628-3p	1/3	0.016	LRP6
6	hsa-miR-5092	1/33	0.018	LRP6
7	hsa-miR-4634	1/42	0.023	LRP10
8	hsa-miR-4774-3p	1/54	0.029	LRP10
9	hsa-miR-4473	1/56	0.030	LRP12
10	hsa-miR-126-3p	1/58	0.031	LRP6
11	hsa-miR-6782-3p	1/58	0.031	LRP10
12	hsa-miR-514b-3p	1/64	0.035	LRP6
13	hsa-miR-514a-3p	1/65	0.035	LRP6
14	hsa-miR-548b-3p	1/77	0.041	LRP10
15	hsa-miR-122-5p	2/61	0.042	LRP3;LRP11
16	hsa-miR-3152-5p	1/8	0.043	LRP10
17	hsa-miR-633	1/81	0.044	LRP12
18	hsa-miR-1277-5p	2/624	0.044	LRP8;LRP6
19	hsa-miR-4511	1/84	0.045	LRP10
20	hsa-miR-3119	1/85	0.046	LRP8
21	hsa-miR-8071	1/87	0.047	LRP10
22	hsa-miR-190a-3p	2/648	0.047	LRP8;LRP6
23	hsa-miR-580-5p	1/89	0.048	LRP6

**Table 5:** Protein-protein interaction network of eleven LRP family members and their ligands using string databases, respectively

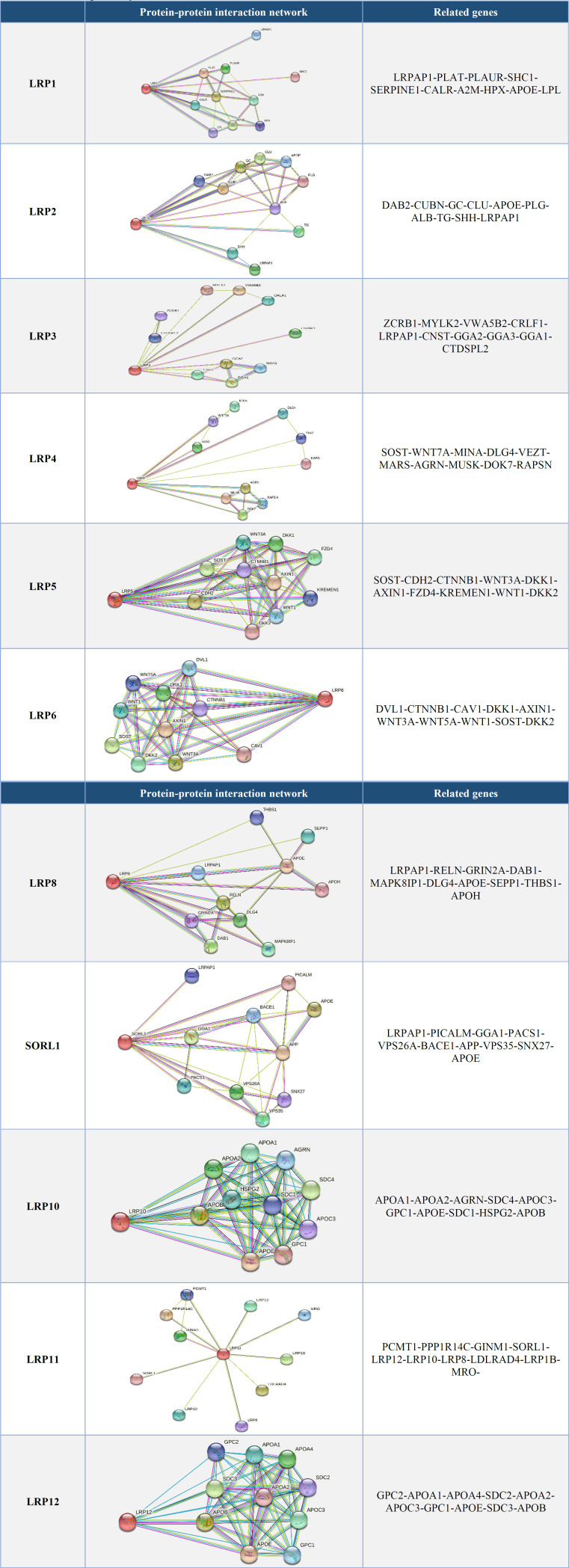

## Discussion

LRP1 relates with more than 30 distinct ligands, such as lipoproteins, viruses, toxins, proteases, growth factors, membrane-anchored and proteins matrix proteins, and internalizes them; it can also start and control signaling pathways. LRP-1 is necessary for vasculature. LRP1 modulates the PDGF signaling pathway by endocytosis of PDGF-R in smooth muscle cells. LRP-1 regulates cell migration and invasion (21). LRP1 and LDLR help improve LDL-C uptake from the blood in BC cells (22). Megalin/LRP2 is a multi-ligand endocytic receptor expressed on some epithelial cells, including the kidney, mammary gland, lung, thyroid, colon, epididymis, prostate, gallbladder, neurons, and macrophages. Its ligands consist of vitamins A, B12, and D and their transporter proteins complexes, apolipoproteins, angiotensin II (Ang II), insulin, leptin, albumin, and connective tissue growth factor (CTGF). TGF-ß decreases megalin levels through the smad2/3 mechanism (23). LRP3 is the most unidentified member of the LRPs family, which might be involved in the signal transduction and/or internalization of lipophilic molecules (24). LRP4 is a receptor for several ligands, like Wnt and Agrin. Like other LRPs, LRP4's role is receptor endocytosis, trafficking, and intracellular signaling. Biologically, LRP4 is elaborate in many progressions, such as kidney development, limb development, neurodevelopment, osteogenesis, and craniofacial organogenesis. Loss of LRP4 in mice outcome in death due to paralysis because of the neuromuscular junction (NMJ) failure to develop, thus avoiding the relation between muscle cells and motor neurons (25). lrp5, 6, 7 have a pivotal activity in the transducer of Wnt ligand pathway signaling. They are coreceptors associated with fizz protein. LRP5 or LRP7 is highly expressed in numerous tissues LRP5 expression is suggested to be related to aptitude for diabetes. The LRP5 gene is associated with bone development and cholesterol metabolism. LRP6 has almost the same structures and functions as LRP5. Like LRP5, it also cooperates with the frizzled (FZD) family, which has seven transmembrane receptors to the Wnt/𝛽-catenin signaling pathway activation. LRP6 has been described in a wide-ranging panel of tumors, such as prostate tumors, breast malignancy, retinoblastoma, and hepatocellular carcinoma. The LRP6 expression was upregulated in these tumors and altered LRP6 leads to abnormal Wnt protein activation. Apolipoprotein E receptor 2 (APOER2) orLRP8, including seven conserved LDL-A repeats followed by 𝛽-propeller motif and 3 EGF receptor-like domains. LRP8 was first recognized in the brain and originated plentifully in ovaries, placenta, and epididymis. LRP8 has also been known as a positive regulator of the Wnt/𝛽-catenin signaling pathway LRP8 has also gained much attention in its function as one of the coreceptors intricate in regulating tumor development (11). LRP9 or sorl1 gene encodes proteins appropriate to at least two families: the LDLR family and the vacuolar protein sorting 10 (VPS10) domain-containing receptor class. The encoded protein also comprises fibronectin type III repeats and an epidermal growth factor repeat. The encoded preproprotein is proteolytically managed to generate the mature receptor, which likely has an essential function in sorting and endocytosis (26). LRP 10 is a likely receptor involved in signal transduction and the internalization of lipophilic molecules and can be uptaken by the lipoprotein APOE in the liver.

Although Wan *et al.* have named BRCA1-associated protein (BRAP) as a tumor suppressor, they could not find such a function for its related genes (27). According to the Human Protein Atlas, LRP 11 is an unfavorable Prognostic marker in breast tumors. LRP 12 is, again, a likely receptor, which might be complicated in the internalization of lipophilic molecules and/or signal transduction. It can act as a tumor suppressor. Grasse *et al.* found LRP12 DNA methylation to be a strong epigenetic prognostic biomarker for carboplatin resistance in non-small cell lung malignancy xenografts (28). In 1999, Qing et al.'s studies first determined a novel putative receptor as an important player in the transformation progression of malignant cells (29). Taheri *et al.* consider the LRP family as a possible biomarker of chemotherapy resistance along with multidrug resistance-associated protein 1 (MRP1) in breast cancer patients (30). According to the Human Protein Atlas, LRP 11 is a disapproving prognostic marker in breast and liver cancer. Also, LRP12 is an unfavorable prognostic marker in endometrial malignancy. In contrast, LRP 10, although it is known as a marker of unfavorable prognosis in liver and kidney cancers, shows inconsistent results in kidney cancer.

## Conclusion

Our study showed that the changed expression of some LDLR members was significantly associated with clinical cancer outcomes in breast cancer patients. However, further investigations are needed to evaluate the studied LDLR members in detail.

## Funding

None.

## Conflict of Interest

The authors declared no conflict of interest.
